# Loose Screws: Removal of Foreign Bodies From the Lower Gastrointestinal Tract

**DOI:** 10.7759/cureus.43093

**Published:** 2023-08-07

**Authors:** John C Hardy, Cody Ashcroft, Carl Kay, Billy-Joe Liane, Christian Horn

**Affiliations:** 1 Internal Medicine, Brooke Army Medical Center, San Antonio, USA; 2 Gastroenterology, Brooke Army Medical Center, San Antonio, USA

**Keywords:** colonoscopy, upper and lower gi tract, ingested foreign body, colonoscopy preparation, foreign body retrieval

## Abstract

While both the American Society for Gastrointestinal Endoscopy (ASGE) and the European Society of Gastrointestinal Endoscopy (ESGE) have released guidelines on the management of ingested foreign bodies in the upper gastrointestinal (GI) tract, neither has addressed indications or techniques for endoscopic removal of foreign bodies in the lower GI tract, perhaps due to the high likelihood of foreign body passage once the foreign body has reached the lower GI tract. We present the case of a 45-year-old woman presenting to the emergency department following the intentional ingestion of multiple screws and nails, complaining of acute abdominal pain and nausea. Imaging revealed four nails in the colon and two adjacent screws in the distal small bowel. In the absence of signs of acute obstruction or bowel perforation, she was admitted for expectant management but subsequently required endoscopic removal of two retained screws in the lower GI tract.

## Introduction

Ingested foreign bodies are a frequently encountered complaint in medicine and can be a significant cause of morbidity and mortality, with annual fatalities being reported as high as 1,500 [1]. Complications related to foreign body ingestion include impactions, perforations, bleeding, and obstruction [2-3]. Fortunately, in approximately 80% of patients, spontaneous passage occurs without complication, especially once the foreign body has reached the lower gastrointestinal tract [4]. However, the applicability of this data (obtained from predominately pediatric case series in the pre-endoscopic era) to the associated guidelines (or lack thereof) is uncertain, and more recent data focused on occurrences in the adult population suggests that the need for endoscopic intervention could be as high as 63%-76% [2,4-6]. This is likely related to the materials ingested and the fact that, in adults, ingestion is commonly intentional and frequently occurs in the setting of comorbid psychiatric disorders [2,6].

While both the American Society for Gastrointestinal Endoscopy (ASGE) and the European Society of Gastrointestinal Endoscopy (ESGE) address endoscopic management of foreign bodies in the upper gastrointestinal (GI) tract, there is minimal guidance on the use of endoscopy when foreign bodies are in the lower gastrointestinal tract [7-8]. In most instances, patients are primarily managed conservatively with serial radiographs to ensure spontaneous passage. If a foreign body fails to pass, surgical consultation is recommended, with time to intervention varying based on the characteristics of the ingested object, as well as the presence of complications. We present a case of foreign body retention in the cecum that was successfully managed by endoscopic retrieval. This article was previously presented as a poster at the 2022 National American College of Gastroenterology Conference on October 23, 2022.

## Case presentation

**Figure 1:**
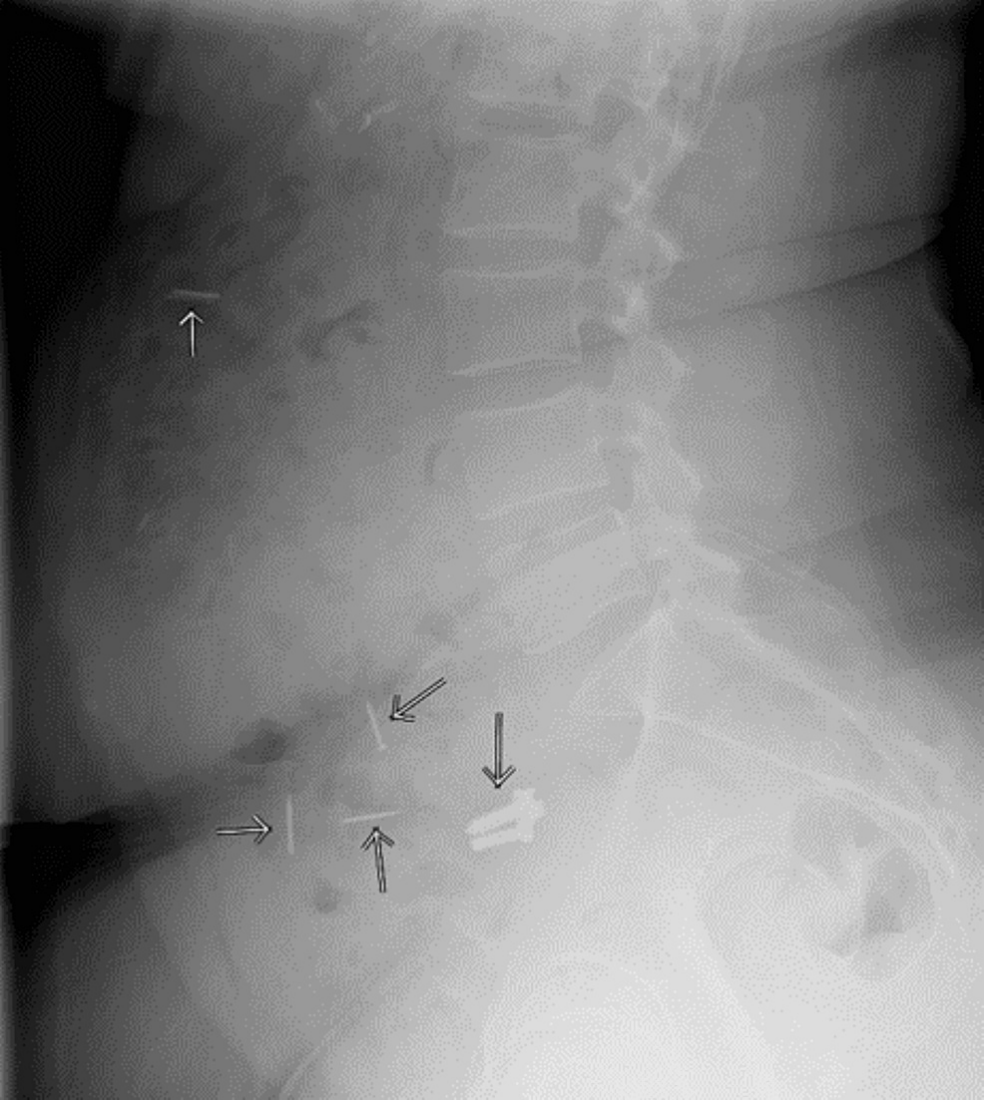
Four nails in the colon and two adjacent screws in the small bowel.

A 45-year-old female with a significant psychiatric history presented to the emergency department with a chief complaint of acute-onset non-localized abdominal pain and nausea. She reported visiting her Rastafarian, who made her a “Tack Shake” to help with her symptoms of anxiety and depression. She reported drinking this mixture the day prior to the presentation. Initial imaging showed four nails in the colon and two adjacent screws in the small bowel (Figure 1), and she was admitted for serial abdominal imaging and monitoring.

**Figure 2:**
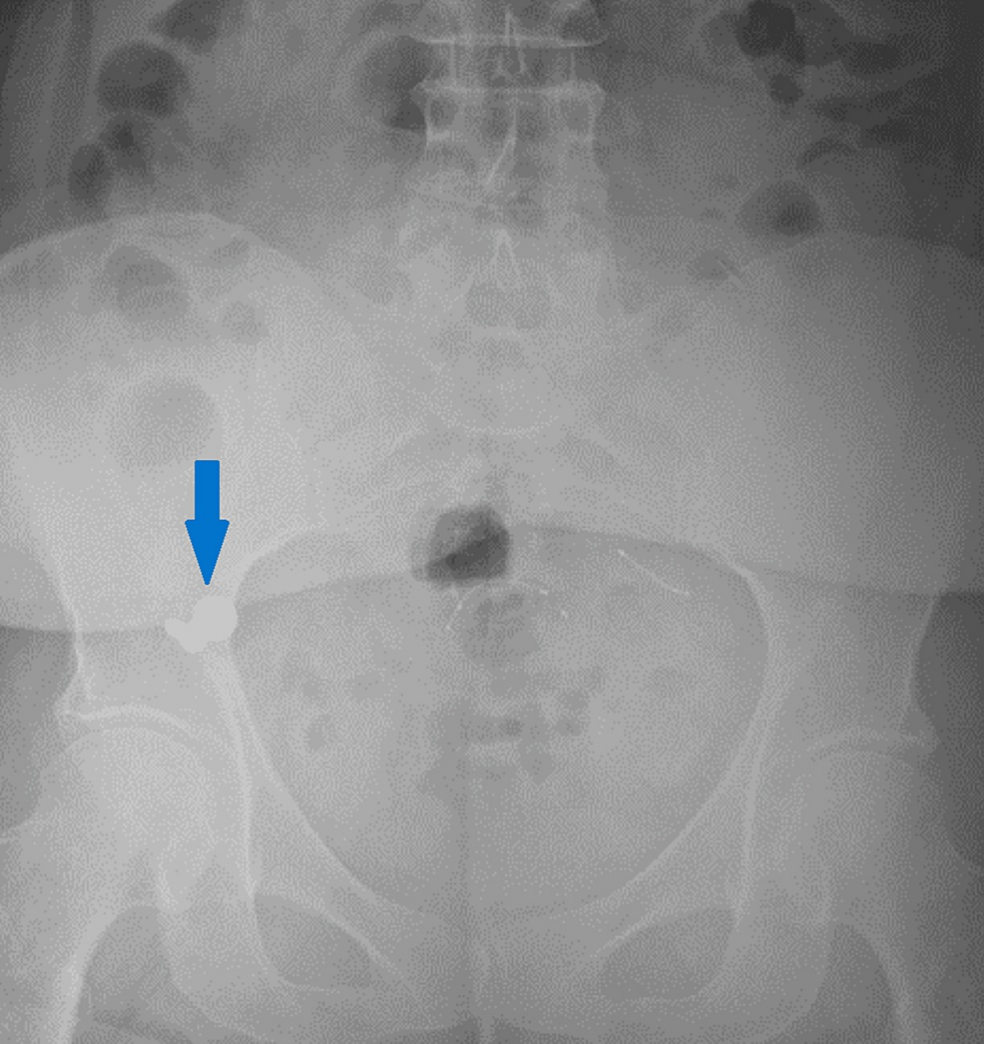
Unchanged positioning of screws in the right lower abdominal quadrant.

After five days and eight liters of bowel preparation, the patient had spontaneously passed all nails, but the screws had not changed position (Figure 2), raising concern for failure to pass the ileocecal valve.

**Figure 3:**
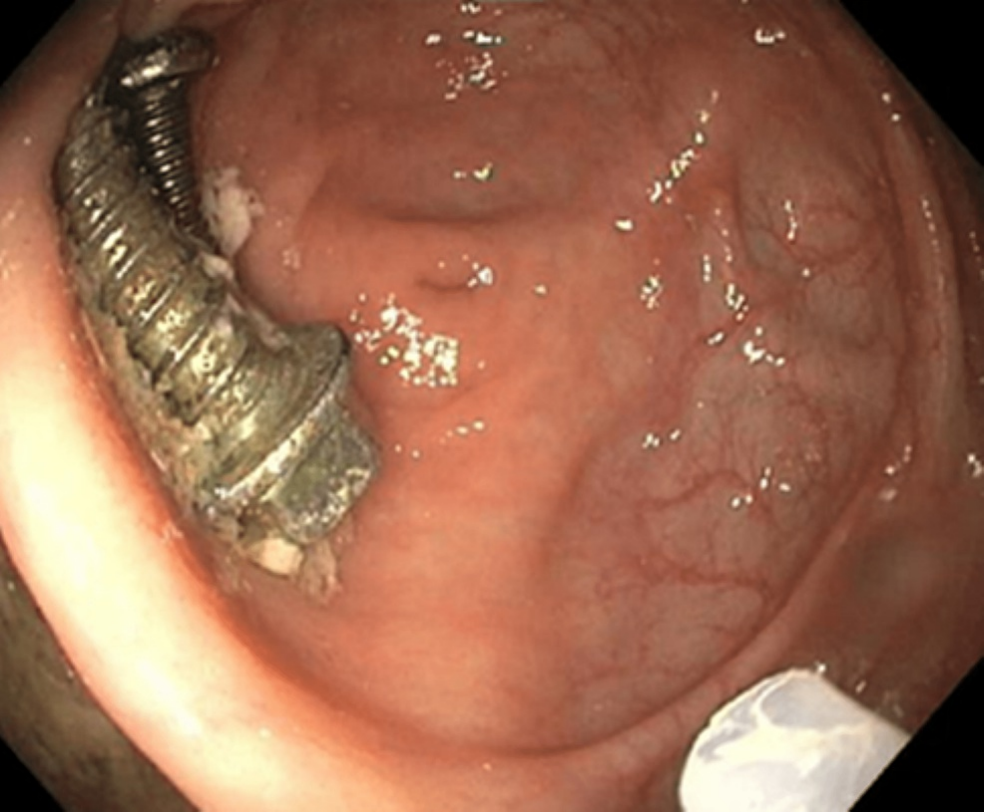
Intertwined screws in the cecum.

An ileocolonoscopy was attempted on hospital day five but failed to locate the screws due to inadequate bowel preparation. Following additional bowel preparation, a second colonoscopy was performed on hospital day six. The screws were located in the cecum and appeared to be intertwined, although they were separated on subsequent manipulation (Figure 3).

**Figure 4:**
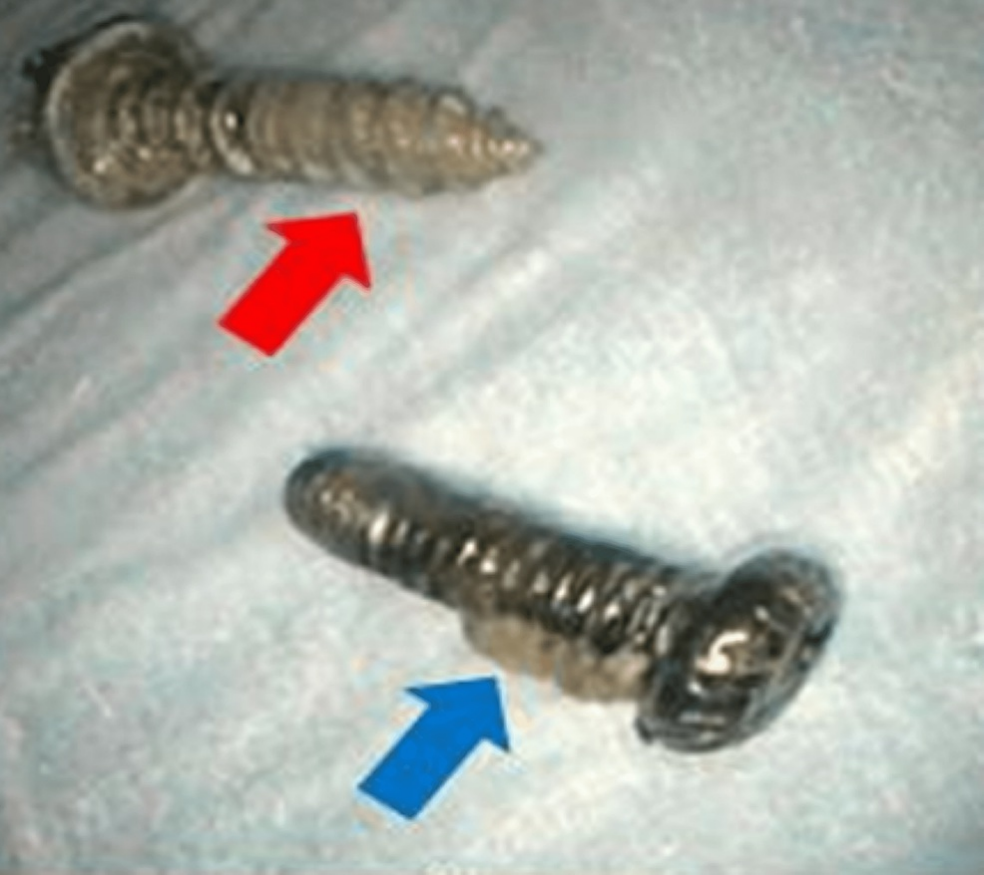
Red arrow: removed sharp screw; and blue arrow: removed blunt screw.

One screw was removed utilizing a Roth Retrieval Net, while the second screw was removed using a cold snare (Figure 4). During retraction, care was taken to orient the sharp end of the screws away from the colonoscope, in an attempt to minimize the risk of injury and perforation.

The patient had an uncomplicated postprocedural recovery. Psychiatry followed the patient throughout her stay and assisted in management. She was ultimately discharged in stable condition to an inpatient psychiatric facility.

## Discussion

Although the majority of both sharp and dull objects will pass spontaneously within four to six days and can be managed with supportive care and observation, the decision to intervene in cases of foreign body ingestion can be challenging [9]. Special care must be taken with foreign bodies longer than 6 cm or more than 2.5 cm in diameter, disk batteries, and magnets, all of which carry varied indications for intervention. Additionally, when sharp objects have been ingested, as in this particular case, endoscopic retrieval is recommended when above the proximal duodenum [5]. Distal to the stomach, surgical intervention is generally considered for foreign bodies that remain in place for greater than one week, although earlier intervention may be considered in special situations to include ingestion of sharp objects or magnets [5].

In this case, the retained screws were in place for six days. Surgical intervention was considered but ultimately deferred in place of lower endoscopy. Although there are no guidelines to support this approach, the size and location of the objects were deemed to be amenable for endoscopic retrieval, which was completed without complication, although notably requiring extensive bowel preparation. While the exact etiology of her initially failed bowel preparation is uncertain, this patient's risk factors for inadequate bowel preparation include her inpatient status and multiple comorbidities/polypharmacy [10].

This patient was ultimately admitted to an inpatient psychiatric facility for suicidal ideation in the setting of multiple comorbid psychiatric disorders. The ingested "tack-shake" at the instruction of her Rastafarian appears to be a previously unreported alternative medicine for anxiety. The authors were unable to locate any prior mention of this practice in the literature, and without evidence of a delusional disorder on psychiatric evaluation, her self-report was presumed to be an honest description.

## Conclusions

This case increases the body of evidence needed to one day formulate guidelines on the appropriate timeline and indications for endoscopic removal of ingested foreign bodies in the lower gastrointestinal tract. It also demonstrates two successful separate methods for the removal of foreign objects by lower endoscopy. Finally, it highlights a potentially new complementary and alternative medicine practice not previously reported in the literature: a drink/mixture called a “Tack Shake/Smoothie,” which contains screws and nails and is purported to assist with anxiety.
